# Tunable Nanoeffect of ZnO on the Properties of Poly(hydroxybutyrate) Membranes

**DOI:** 10.1021/acsomega.5c08548

**Published:** 2025-12-03

**Authors:** Tainan Miguel, Winnie Queiroz Brandão, Martinho Rau, Michele Debiasi Alberton, Ivonete O. Barcellos, Lizandra Maria Zimmermann

**Affiliations:** † Department of Chemistry, 37874 Regional University of Blumenau—FURB, 89030-903 Blumenau, SC, Brazil; ‡ Department of Pharmaceutical Sciences, 37874 Regional University of Blumenau—FURB, 89030-903 Blumenau, SC, Brazil

## Abstract

Polymeric membranes composed of poly­(3-hydroxybutyrate) (PHB) integrated with zinc oxide nanoparticles (ZnO NPs) or quantum dots (ZnO QDs) were successfully prepared by using a sol–gel method for nanostructure dispersion. The resulting PHB/ZnO membranes exhibited uniform morphology and fluorescence with well-dispersed nanostructures confirmed by UV–vis spectrophotometry, fluorescence analysis, and FTIR spectroscopy. Spectral analysis revealed that ZnO NPs and QDs enhanced UV-blocking capabilities and reduced the level of membrane yellowing. Physicochemical evaluations showed that the fluorescence remained stable under prolonged water vapor exposure. Increasing the ZnO content (5, 10, and 15%) led to higher membrane density, wettability, and hardness. Swelling and water vapor permeability (WVP) tests indicated that ZnO QDs were the most effective in reducing moisture diffusion, suggesting the formation of internal barriers. Despite increased surface hydrophilicity, WVP decreased, highlighting the dual functionality of the nanostructures. Antibacterial assays demonstrated inhibition of *Staphylococcus aureus* growth by up to 30.08% (ZnO NPs) and 21.83% (ZnO QDs). These findings support the potential of PHB/ZnO membranes as multifunctional materials for advanced packaging applications, offering tailored UV protection, moisture control, and antimicrobial properties suitable for the food, pharmaceutical, and cosmetic industries.

## Graphical abstract

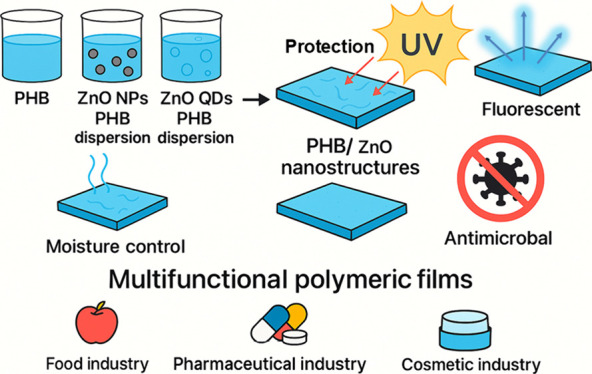

## 1 Introduction

The widespread use of plastics across various industries has intensified the challenges associated with their extensive application, particularly in single-use scenarios.^1^ Their chemical stability means they can persist for centuries, and their breakdown into smaller particles poses risks to both terrestrial and marine ecosystems. Improper disposal of plastics has far-reaching environmental, economic, and social consequences.^2,3^ The scientific community and many social and industrial organizations are exploring key solutions such as reducing plastic consumption impact, promoting recycling efforts, circular economy practices, extending the lifespan of plastic products, and creating new solutions to replace conventional plastic usage.^1,4^ Biobased plastics are derived from renewable biological sources such as plants and biomass, but are not necessarily biodegradable or compostable. In contrast, biodegradable plastics are designed to break down through natural microbial activity, converting into water, carbon dioxide, and biomass, although the rate and conditions for degradation vary. Compostable plastics are a subset of biodegradable plastics that specifically decompose under controlled composting conditions, leaving no toxic residue, as assigned by ASTM D6400.^5^ While all compostable plastics are biodegradable, not all biodegradable plastics meet the stringent criteria for compostability.^6^ Biodegradable polymers usually come from a variety of bioresources, such as wastes of food, coproducts from agro-industrial processes, and other sources such as starch and cellulose. In addition to their biodegradability, many polymers are notable for their multifunctionality, possessing a range of biological, physical, and chemical properties.^7^

Polyhydroxyalkanoates (PHAs) are biodegradable biopolymers biosynthesized by various microorganisms’ genes and substrates as carbon sources like glucose, fatty acids, and waste materials. Additionally, PHA production is being optimized using renewable carbon feedstocks, including agricultural waste, food waste, and wastewater, to lower costs and improve sustainability. Emerging approaches involve the use of transgenic plants to produce PHAs, leveraging genetic modifications to enhance the biosynthetic yield in crops. PHAs encompass a diverse family of polymers, including poly­(3-hydroxybutyrate) (PHB) and poly­(3-hydroxybutyrate-*co*-3-hydroxyvalerate) (PHBV), which offer promising alternatives to petroleum-based plastics due to their biocompatibility and environmental degradability. They can be broken down aerobically and anaerobically without requiring specific environmental conditions, making them suitable for applications in medical, packaging, and agricultural sectors.^7^ PHB is a thermoplastic polyester with a high melting temperature (160–180 °C), a high degree of crystallinity, with hydrophobic properties, that is, low permeability for O_2_, H_2_O, and CO_2_, in addition to being compatible with the human body, expanding the possibilities of applications.^7–10^ However, PHB has many drawbacks, including stiffness, brittleness, and low thermal stability.^10^ PHB lacks intrinsic antimicrobial properties, which points to the need to develop composites with additives that introduce new functionalities and improve its performance.^11^

Given growing environmental concerns, zinc oxide (ZnO) nanoparticles (NPs) and quantum dots (QDs) are particularly attractive due to their low toxicity, making them environmentally friendly and suitable for a wide range of applications.^12–15^ While both NPs and QDs are nanoscale materials, they differ primarily in size and in the effects of quantum confinement. ZnO QDs exhibit quantum confinement in all three dimensions, allowing their optical properties to be tuned based on particle size, especially when their dimensions approach the exciton Bohr radius.^16^ In contrast, ZnO NPs generally range from a few nanometers up to hundreds of nanometers. These NPs have been reported in various sizes and morphologies, with properties depending on those investigated properties, as the case of ZnO at the nanoscale being reported, to act as antibacterial agents,^17,18^ UV blocker,^19^ and photocatalyst.^20–23^ The photoprotection characteristic of ZnO is very important in the production of materials with greater durability, resistance, and high conservation.^24^

Studies have developed sustainable PHB-ZnO composites for biodegradable 3D printing filaments. ZnO enhances PHB’s thermal stability, alters crystallinity, and improves processability. Chemical interactions between ZnO and PHB modify the polymer structure, increasing the amorphous characteristics suitable for extrusion. Morphological analysis confirms good ZnO dispersion, and PHB-0.5%ZnO has been successfully printed into scaffolds, showing promise for biomedical and packaging applications.^25^

Another study developed eco-friendly ZnO/PHB composites with enhanced antibacterial and biodegradable properties. Using a simple casting method, green-synthesized ZnO microporous particles were uniformly dispersed in PHB films, significantly improving antibacterial and antibiofouling performance. Under LED light exposure, the composites achieved bacterial inactivation rates of 97.5% against *Escherichia coli* and 76.2% against *Staphylococcus epidermidis*, outperforming commercial ZnO NPs. Additionally, the films exhibited rapid biodegradation with 99% decomposition in soil within 10 weeks. These findings position PHB-ZnO composites as cost-effective and sustainable solutions for antibacterial packaging.^11^

This study introduces a novel approach by systematically investigating the nanoeffect of ZnO, from QDs exhibiting quantum confinement to NPs, on the physicochemical properties and functional performance of PHB-based nanocomposites. Unlike previous studies that focused on either ZnO NPs or QDs in isolation, this work explores the synergistic and size-dependent interactions between ZnO and PHB, aiming to tailor material properties for enhanced biodegradability, antimicrobial activities, and photostability. By bridging the gap between quantum-scale and nanoscale ZnO, the research offers new insights into optimizing PHB composites for advanced applications in sustainable, for instance, packaging and biomedical materials.

## 2 Materials and Methods

### 2.1 Materials

Zinc acetate dihydrate ACS 98% was purchased from Sigma-Aldrich (USA). Sodium hydroxide, potassium hydroxide ACS reagent >98%, and ethanol ACS 99.5% were supplied by Vetec (Brazil). Chloroform ACS > 99.8%, poly­(3-hydroxybutyrate) (PHB) was kindly supplied by PHB Industrial S.A. (Brazil) with a molecular weight of 115,000 g mol^–1^, was obtained from sugar cane fermentation, registered under the brand name BIOCYCLE with 3.14% of the nominal content of estradiol valerate and 1000 mm granulometry (30–18 mesh). Astrazon Blue FGGL 300% was kindly supplied by Dystar (Brazil). Brain-heart infusion broth, Mueller-Hinton agar, and broth were purchased from Kasvi (Brazil) for the antimicrobial tests. All reagents were used without further preparation or purification.

### 2.2 Synthesis of ZnO NPs and ZnO QDs

The synthesis of ZnO NPs was performed according to a protocol previously described in the literature.^26^ Initially, two solutions were prepared separately: 0.645 g of zinc acetate dihydrate and 0.232 g of potassium hydroxide were dissolved in 27.5 and 12.5 mL of ethanol, respectively. The solutions were then stirred in a water bath at 60 °C until they were completely dissolved. The zinc acetate dihydrate solution was then placed in a round-bottomed flask, followed by a slow drip of the potassium hydroxide solution (the timer was started after the first drop). The reaction was performed under constant stirring in a water bath at 60 °C for 2 h. The final concentrations in the reactor were 7.3 × 10^–2^ and 1.0 × 10^–1^ mol L^–1^ for zinc acetate and potassium hydroxide, respectively. After 2 h, the NPs dispersion was divided and distributed into 4 tubes (10 mL each) and centrifuged for 15 min. Finally, the sediment was redispersed in 10 mL of ethanol and washed twice.

ZnO QDs were synthesized as described previously by Zimmermann and co-workers.^21^ The protocol consisted of first preparing 10 mL of two stock solutions in ethanol: zinc acetate dihydrate at 4.5 × 10^–3^ mol L^–1^ and sodium hydroxide at 0.02 mol L^–1^. Both solutions were heated in a water bath at 60 °C with constant stirring until they were entirely homogenized. Subsequently, 7.1 mL of ethanol was added to a reaction flask, followed by 6.6 mL of the zinc acetate stock solution. This mixture was kept in an ice bath (0 °C) for 10 min with magnetic stirring, when 1.3 mL of sodium hydroxide was slowly dropped into the flask to start the formation of QDs (the timer was started at the first drop). After 90 s of reaction time, the system was transferred to a water bath at 60 °C, where it was stirred for 2 h. The final concentrations of zinc acetate and sodium hydroxide were 2.0 × 10^–3^ and 2.5 × 10^–3^ mol L^–1^, respectively.

The precipitation of QDs was based on the method already described in the literature by Jacobsson et al.^27^ After 2 h of QD synthesis, the volume was transferred to an Erlenmeyer and 50 mL of hexane was added. The resulting cloudy dispersion was stirred at room temperature for 24 h. After this period, the dispersion was centrifuged for 30 min, and the particles were sedimented. The sediment was redispersed in 2 drops of ethanol and washed twice.

### 2.3 Preparation of PHB/ZnO NPs and PHB/ZnO QDs Membranes

The modified PHB membranes were prepared according to what was previously reported,^28^ with minor modifications. To prepare the PHB membrane with ZnO NPs (PHB/ZnO NPs), 0.08 g of PHB was initially added to 8 mL of chloroform in an ultrasonic bath for 2 h at 50 °C to ensure complete dissolution of PHB. Then, the NPs sediment (varying from 5%, 10%, and 15% in mass), previously prepared (Section 2.2), were redispersed in chloroform and added to the PHB solution, which was immediately transferred to a glass Petri dish. Finally, the dispersion was kept in the hood for 24 h at room temperature for complete solvent evaporation and formation of the PHB/ZnO NPs membrane. The preparation of the PHB/ZnO QDs membrane occurred similarly to the procedure described for the NPs. The neat PHB membrane was also prepared for comparison.

### 2.4 Characterization of NPs and QDs Dispersions

#### 2.4.1 Spectroscopic Characterization and Dynamic Light Scattering

The ultraviolet–visible (UV–vis) absorption spectra of ZnO NPs and QDs were measured with a UV-1800 spectrophotometer (Shimadzu, Japan) in the 200–400 nm range. This characterization allowed the observation of quantum confinement in the semiconductor particles, the determination of particle size, and the value of the band gap energy, the latter two being calculated with eqs S1 and S2, respectively. The hydrodynamic size of the NPs and QDs was measured by the dynamic light scattering (DLS) technique using a Zetasizer Nano ZS instrument (Malvern Panalytical, U.K.). DLS measurements were performed in triplicate, considering the average value.

#### 2.4.2 Transmission Electron Microscopy

Transmission electron microscopy (TEM) analyses were performed using a JEOL JEM 1200EX-II TEM (Electron Microscopy Center (CME)/UFPR), which operates at an accelerating voltage of 120 kV. A 10 μL portion of the samples (NPs dispersion) was dropped on a copper grid with an ultrathin carbon film. The authors previously reported TEM images for ZnO QDs.^21^

### 2.5 Characterization of PHB/ZnO NPs and PHB/ZnO QDs Membranes

#### 2.5.1 Spectroscopic Characterization

UV–vis absorption spectra of the membranes were investigated in the wavelength range of 700–200 nm using a PerkinElmer (USA) Lambda 25 UV–vis spectrophotometer coupled to an integrating sphere. The fluorescence emission spectra (excitation wavelength (λ_exc_) of 330 nm) were recorded with a Cary Eclipse spectrofluorometer (Agilent, USA) equipped with a xenon lamp in the 300–800 nm range. The Fourier transform infrared (FTIR) spectra of the membranes were evaluated in the range of 4000–500 cm^–1^ using a Bruker (USA) Vertex 70 Platinum spectrophotometer in attenuated total reflectance (ATR) mode. This technique also allowed the evaluation of membranes exposed to UV light (26 W) for 45 days (the period required to detect a significant change in the FTIR spectrum^29^). Quantification was assessed by the intensity ratio method between the characteristic bands of the constituents of the membrane.

A remission spectrophotometer (CM-3610d, Konica Minolta—Japan) was used to perform colorimetric analysis of the membranes. The color change was evaluated after 45 days of UV irradiation using Taschibra TKT 26-2 RGG 26 W, 50/60 Hz, placed inside a black box. The measurements were carried out in the visible spectral range (400–700 nm), and the color intensity (*K*/*S*) was determined from the reflectance values using the Kubelka–Munk equation (eq 1).^30,31^

$$\frac{K}{S}=\frac{(1-R{)}^{2}}{2R}$$ 1

where *K* is the absorption, *S* is the scattering coefficient of the examined membranes, and *R* is the measured reflectivity signal. The residual color difference (Δ*E*) was calculated from eq 2.^32^

$$\Delta E=\sqrt{(\Delta H{)}^{2}+(\Delta C{)}^{2}+(\Delta L{)}^{2}}$$ 2

where Δ*H* corresponds to the tonality deviation, Δ*C* to the purity deviation, and Δ*L* to the lightness deviation, all parameters listed in the equations were determined by using the remission spectrophotometer.

#### 2.5.2 Physicochemical Analysis

The visual fluorescence emission stability of the PHB membrane with 15% ZnO NPs was evaluated in the presence of water vapor. The membrane was first weighed and kept in a completely closed system for 180 days to verify whether ambient humidity affected the fluorescence stability. During this period, 1 mL of distilled water in the system was replaced three times. After 180 days, the membrane fluorescence was evaluated by UV light irradiation (excitation wavelength of 365 nm) and weighing to verify possible gains or losses in mass during the experiment.

The density of the neat PHB membranes and those modified with nanostructures was determined by the ratio between the mass (g) and volume (cm^3^) (eq S3). The hardness data of the membranes were collected from tests with a Shore A durometer (ASTM D 2240). The procedure consisted of pressing the indenter against the sample for 10 s at three random points in the material. After the data were recorded, the arithmetic mean of the hardness values was calculated.

The swelling index, expressed as a percentage (% *I*), of the PHB, PHB/ZnO NPs, and PHB/ZnO QDs (15%) membranes, was determined by comparing their masses before (*m*_Dry_) and after (*m*_wet_) immersion in water. First, each membrane sample was placed in an oven at 70 °C for 24 h to remove moisture, and then, its mass was determined. After that, the dried membrane was immersed in water at room temperature and removed at specific time intervals (12 and 24 h) to monitor the mass gain. The membranes’ swelling index was calculated with eq 3.^33^

$$\%I=\frac{m_{\mathrm{wet}}-m_{\mathrm{Dry}}}{m_{\mathrm{Dry}}}\times 100$$ 3

where *m*_Dry_ and *m*_wet_ are the dry and wet membrane mass, respectively.

The diaphragm cell method was used to analyze the ability of membranes to permeate water vapor. For this purpose, a permeation cell with a desiccant (silica gel) was placed in a desiccator under controlled humidity conditions. In this experiment, the permeation cell with a membrane with a known thickness and mass of neat PHB and PHB with 5% and 15% ZnO NPs and QDs. The materials were subjected to pressure cycling between two specified surfaces at a relative humidity of 57–60% and a temperature of 20 °C. The water vapor permeability (WVP) was calculated using eq 4.^9^

$$[\mathrm{WVP}]=\frac{m_{H_{2}O}\times x}{A}$$ 4

where [WVP] = water vapor permeability (g mm cm^–2^); *m*_H_2_O_ = amount of water absorbed (g) by the sample; *x* = membrane thickness (mm); *A* = area of the sample contact surface (cm^2^).

The wettability was investigated by measuring the contact angles of PHB and PHB membranes with ZnO NPs and QDs. In this experiment, 10 μL of distilled water was deposited on the membranes at three different locations, and the droplet formed was captured after 1 and 8 min using a USB digital microscope at a magnification of up to 1000×. The ImageJ program measured the contact angle.

All statistical analyses were performed through analysis of variance (ANOVA, between subjects) via Fisher’s tests, using Statistica software version 14 (TIBCO Software Inc.). *P-*values below 0.05 were considered statistically significant with a 95% confidence level.

#### 2.5.3 Thermogravimetric Analysis

Thermogravimetric analysis (TGA) measurements were carried out using a PerkinElmer 4000 instrument under a synthetic air atmosphere at a flow rate of 20 mL min^–1^. The temperature was ramped up from 30 to 800 °C at a heating rate of 10 °C min^–1^.

### 2.6 Applications of Composite Membranes

#### 2.6.1 Antibacterial Properties

The potential for inhibition of bacterial growth by PHB and PHB with ZnO NPs and ZnO QDs membranes was investigated against Gram-positive bacteria (*Staphylococcus aureus* (ATCC 25923)), following the protocol already presented in the literature.^34^ Microorganisms were grown in BHI broth for 24 h at 37 °C and then plated on Mueller–Hinton agar (incubated for 24 h at 37 °C) to reactivate the bacterial strains. The bacterial inoculum was prepared by suspending the colonies in phosphate buffer (pH 7.0) and adjusting to McFarland scale 0.5 (5.0 × 10^8^ CFU mL^–1^). Then, 5 mL of the inoculum was transferred to 45 mL of Mueller–Hinton broth. From this mixture, 1 mL was added to sterile tubes containing the membranes of neat PHB and PHB with 5% and 15% of ZnO NPs and ZnO QDs (average mass ∼ 2 mg), previously autoclaved. The tubes were incubated at 37 °C for 24 h. Negative control was included in each test for comparison without the presence of the membranes. Absorbances of samples and negative control tubes were taken at 620 nm, at 0 and 24 h. The percentage of inhibition was calculated using eq 5.

$$\%\;\mathrm{Inhibition}=100-[(\frac{\mathrm{Sample}\;\mathrm{absorbance}}{\mathrm{Negative}\;\mathrm{control}\;\mathrm{absorbance}})\times 100]$$ 5

#### 2.6.2 Photocatalysis

The photocatalytic potential of the PHB membrane incorporated with 15% ZnO NPs was investigated for the degradation of the Astrazon Blue FGGL 300% (AB) dye. The experiments were performed using a jacketed reactor for water circulation and keeping the temperature at 23 ± 1 °C with a sunlight lamp (300 W, OSRAM, GmbH) 9.5 cm away from the dye + PHB membrane/15% ZnO NPs sample. The required volume of AB dye solutions at concentrations of 1.5 × 10^–5^ and 7.5 × 10^–6^ mol L^–1^ was added to the jacketed reactor, and then the membrane was immersed in the solution. The experimental apparatus was placed in a box covered internally with aluminum foil to maximize light reflection toward the sample. The dye solution with the membrane was stirred constantly at room temperature. At predetermined intervals (10 min in the dark, 2, 3, and 4 h), aliquots were collected and analyzed using a full UV–vis spectrophotometric scan, focusing on the absorbance band of AB at λ_max_ = 609 nm. This procedure enabled the monitoring of the photocatalytic degradation percentages. To assess whether light exposure alone influenced the discoloration of the organic molecule, a control experiment was conducted under the same conditions but without the presence of the membrane in the solution. The degradation percentages (%*D*) were calculated using eq 6.

$$\%D=\frac{{\mathrm{ABS}}_{i}-{\mathrm{ABS}}_{f}}{{\mathrm{ABS}}_{i}}$$ 6

where ABS_i_ and ABS_f_ are the initial and final absorbance values, respectively.

## 3 Results and Discussion

### 3.1 Characterization of Dispersions

#### 3.1.1 UV–Vis Spectroscopy and DLS

UV–vis and PL (photoluminescence) spectra of ZnO NPs dispersion are shown in Figure 1a, just after the synthesis time of 2 h. It is possible to observe from the UV–vis spectrum that a dispersed NP system was obtained, as verified by the tangency of the onset of the absorption band in the lower energy region (near 369 nm). Therefore, the particles have sizes greater than the threshold required to exhibit the quantum confinement effect; they do not behave as QDs. This threshold is characterized by a band gap greater than 3.37 eV, which results in absorption starting at 367 nm. By using eqs S1 and S2, the diameter and band gap energy (*E*_g_) of the NPs were determined to be 5.32 nm and 3.37 eV, respectively.

**Figure 1:**
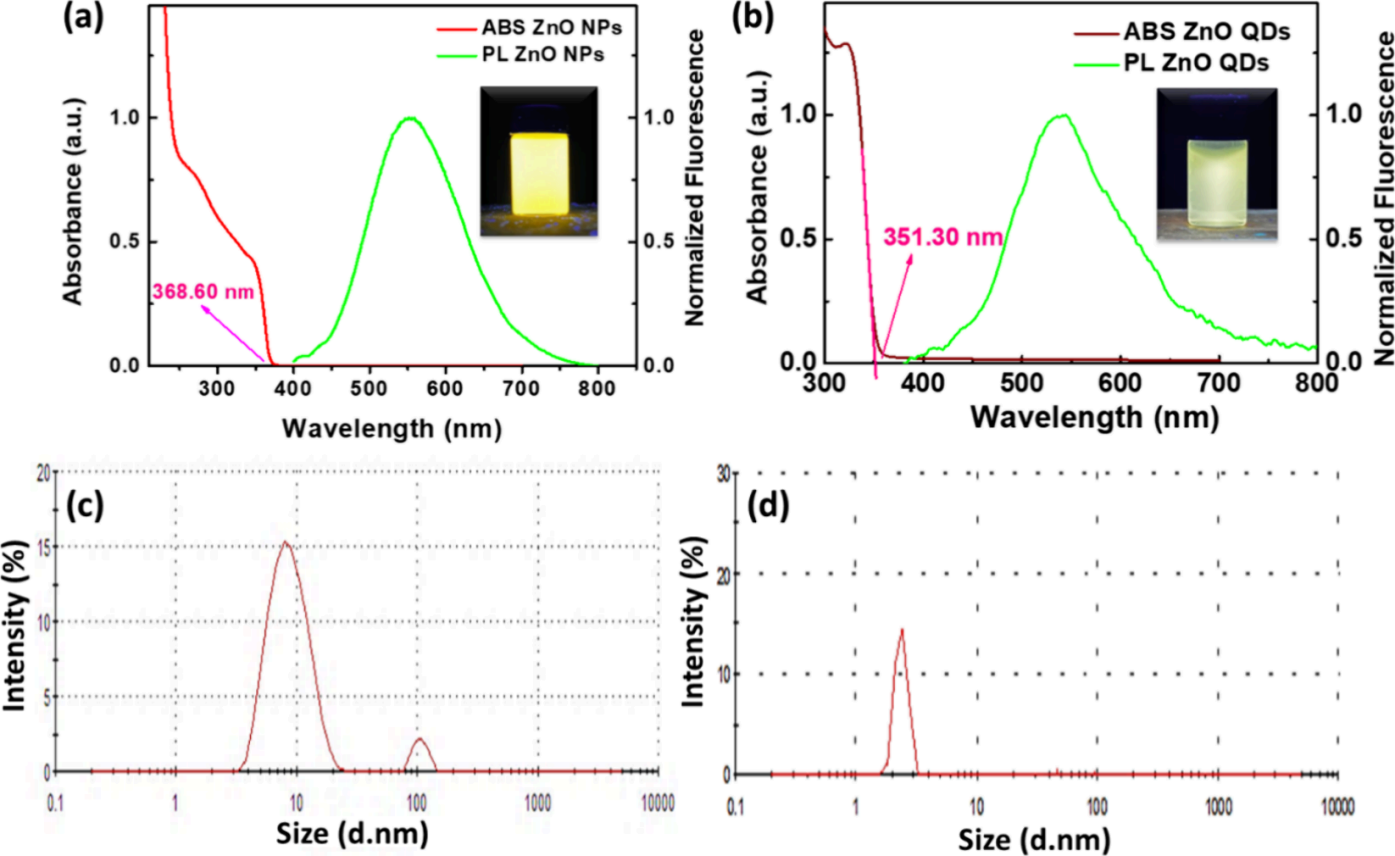
UV–vis spectra and PL emission (λ_ex_ = 330 nm) of the ethanolic dispersion of ZnO NPs (a) and ZnO QDs (b). DLS results for ZnO NPs (c) and ZnO QDs (d). Photographs of NPs and QDs dispersions under UV light irradiation with λ_exc_ = 365 nm are shown in the insets of Figure 1a,b, respectively.

Figure 1b shows the UV–vis and PL spectra obtained for the ZnO QDs dispersion. From the UV–vis spectrum, the onset of the absorption band at the lower energy region (351.3 nm) was used to determine the QDs diameter and band gap energy (*E*_g_), yielding values of 4.10 nm and 3.53 eV, respectively. Visual fluorescence emission under UV lamp excitation (λ_exc_ = 365 nm) was confirmed: the NPs exhibited a yellowish emission, while the QDs showed a greenish emission (inset of Figure 1a,b). Also, as observed by PL emission, there is a red shift from the emission band of ZnO QDs (λ_max_ = 538 nm) to the band emission of ZnO NPs (λ_max_ = 550 nm).

The DLS technique allowed the determination of the hydrodynamic size of the NPs. Figure 1c shows the size distribution of the dispersed particles, indicating an average size of 10 nm and a minor contribution of aggregates, with an average size of 100 nm. The results indicate the reduced particle sizes of colloidal dispersion and the tendency for the material to aggregate without a stabilizing agent. On the other hand, Figure 1d shows the size distribution of the ZnO QDs, where an average size of 4.3 nm was obtained without aggregates. Additionally, DLS measurements were performed to evaluate the aggregation behavior of QDs over time in stored dispersions (Table S1). The analysis indicated that aggregation was significant during the investigated period, with hydrodynamic diameters reaching approximately 15 nm after 21 days. Previous studies using spectroscopic techniques examined the effect of the stabilizer ethylene glycol on ZnO QD dispersions over a 40-day period. These studies confirmed that, in the absence of a stabilizer, the particles tend to agglomerate.^35^ Therefore, it is important to highlight the role of sedimentation and immediate redispersion of the NPs into a polymeric matrix after their preparation, as this procedure tends to minimize the aggregation effects commonly observed in colloidal dispersions.

### 3.2 Shape and Size—TEM

Figure 2 presents a TEM image of the ZnO NPs. The analysis reveals that the mean diameter of the particles is 6.26 ± 0.03 nm, exhibiting a spherical shape. The particle size measured by microscopy is smaller than that determined by DLS, which is anticipated, as DLS typically measures the hydrodynamic diameter of particles in liquid dispersions, accounting for the solvation layer surrounding the particles and being highly sensitive to aggregation. In contrast, TEM provides a more precise measurement of the actual particle size in the dry state, and it is still possible to differentiate the particle boundaries in aggregates. Therefore, the observed discrepancy between the two techniques aligns with the typical behavior of NPs under different analytical conditions.

**Figure 2:**
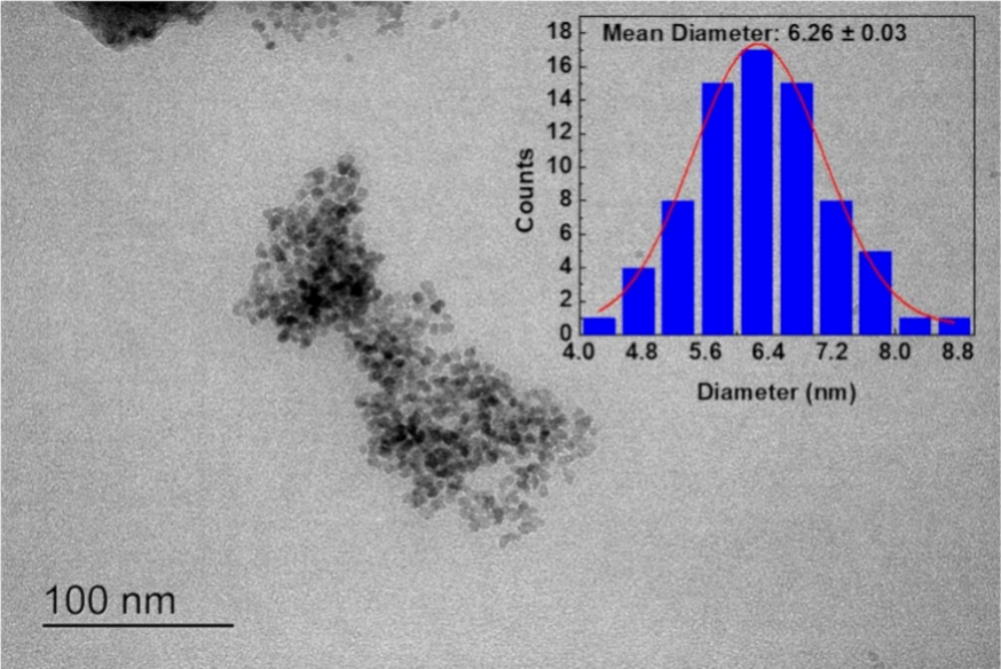
TEM image for ZnO NPs: magnification 50 kx.

In a primary study, the size of ZnO QDs was evaluated by high-resolution transmission electron microscopy (HRTEM), giving results of nanocrystals with an average size = 4.3 ± 0.1 nm. In the same work, by HRTEM, ZnO QDs showed crystalline planes compatible with (100) and (002) of the crystalline structure of hexagonal wurtzite.^21^ Another previous study using the same ZnO QDs synthesis method as presented here, reported, through density functional theory (DFT) calculations, a theoretical particle size of 4.69 nm. This value is in good agreement with HRTEM measurements from that study, which showed average sizes close to 4 nm.^36^

### 3.3 Preparation and Characterization of Membranes

Studies involving the combination of NPs and QDs in polymeric matrices have gained special visibility. The synergistic properties presented by these composite materials make them promising for several applications.^37,38^ In this study, ZnO NPs and QDs were incorporated into the polymer matrix of the PHB membrane; the results are shown in Figure 3. The composite membranes have a nonbrittle appearance, are visibly homogeneous, with the nanostructures uniformly dispersed. Figure 3a shows the PHB membrane under white light, while Figure 3b,c shows those with ZnO NPs and QDs, respectively, under UV light (excitation wavelength (λ_exc_) = 365 nm). As can be seen, the fluorescence emission is yellowish in the case of the membrane with NPs and slightly greenish in the case of the membrane with QDs.

**Figure 3:**
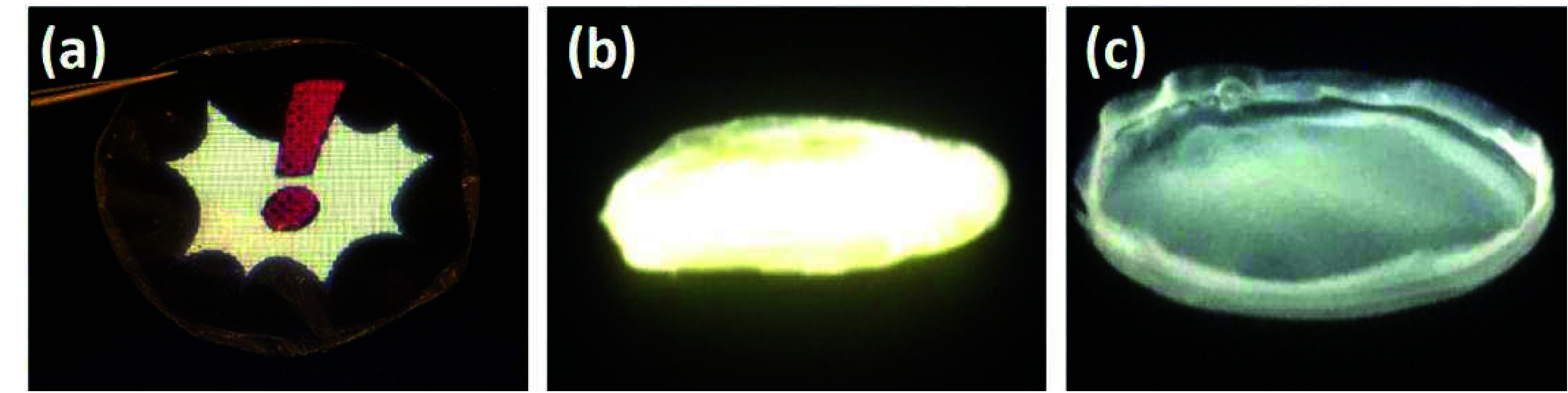
Photograph of the neat PHB membrane under white light (a), the PHB membrane with ZnO NPs (b), and with QDs (c), the latter under a UV light lamp with λ_exc_ = 365 nm.

#### 3.3.1 UV–Vis and Fluorescence Spectroscopy

The incorporation of ZnO NPs and QDs into the PHB membrane was confirmed by UV–vis spectrophotometry. In Figure 4a,b, an absorbance band at 360 nm,^39^ characteristic of the broad absorption spectrum of this semiconductor, can be observed, which does not occur in the case of the spectrum for PHB. As the proportion of NPs and QDs increases, the absorbance intensity rises. There was more significant light scattering for the proportion of 15% NPs due to the possible agglomeration of NPs on the membrane surface. The response demonstrates an improvement in the UV-blocking properties of the PHB nanocomposite with ZnO NPs and QDs, meeting one of the purposes for the formation of the nanocomposite.

**Figure 4:**
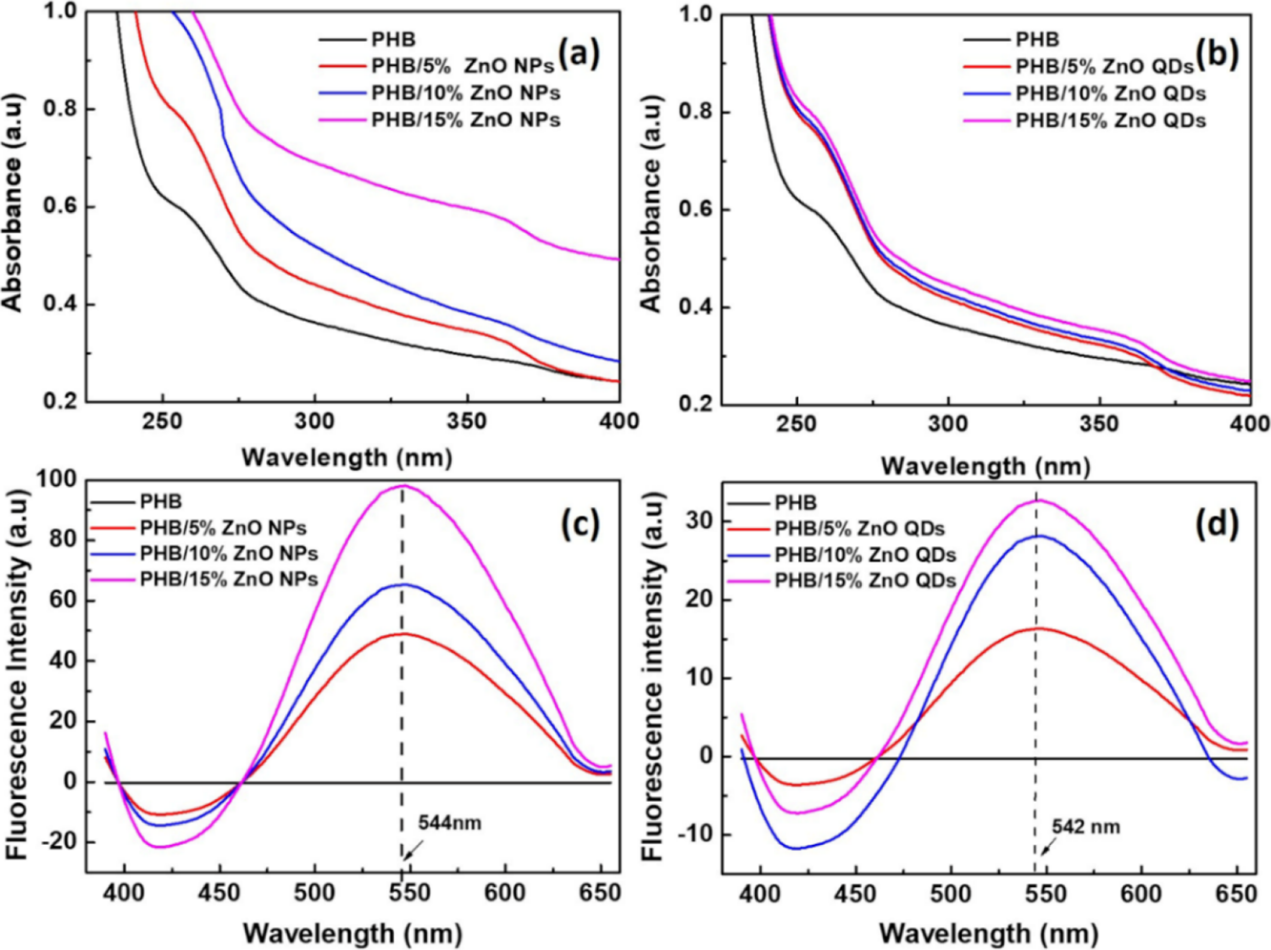
UV–vis and fluorescence emission spectra of PHB membranes with different proportions of NPs (a,c) and ZnO QDs (b,d).

The fluorescence measurements in the PHB and PHB membranes with different amounts of ZnO NPs and QDs (5, 10, and 15%) were performed at the excitation wavelength of 330 nm, chosen based on the maximum absorption wavelength of the ZnO nanostructures. The emission spectra are presented in Figure 4c for PHB/ZnO NPs (emission wavelength (λ_emis_) = 544 nm) and in Figure 4d for PHB/ZnO QDs (λ_emis_ = 542 nm). As observed, in both cases, the increase in the percentage of ZnO NPs and QDs in the PHB membrane increased the fluorescence intensity. As observed, in both cases, increasing the percentage of ZnO NPs and QDs in the PHB membrane led to an enhancement in the fluorescence intensity. The slight redshift in the maximum fluorescence emission wavelength for membranes containing NPs, as corroborated by UV–vis absorbance and PL measurements of dispersions (Figure 1), highlights the distinct optical behavior of QDs. This reinforces their classification as a special type of NP with size-dependent optical properties.

#### 3.3.2 FTIR Spectroscopy Spectra

FTIR also confirmed the addition of NPs and QDs to the PHB polymer matrix. Figure 5a,b shows the FTIR spectra for the PHB membranes with different proportions of NPs and QDs, respectively. The spectra of neat PHB and ZnO are also shown in both cases. The PHB spectrum is characterized by bands of carbonyl group (C=O) at 1718 cm^–1^, the symmetric stretching vibration of C–O–C is indicated at 1274 cm^–1^, in addition to the bands between 1452 and 1378 cm^–1^ due to the asymmetric and symmetric angular deformations of the −CH_3_ groups, respectively, and finally, the C–H stretching vibration of the alkanes is observed in 2980 cm^–1^.^40–42^ Zn–O stretching vibrations are observed at 514 cm^–1^.^43^ As the ZnO content increases, particularly in the case of ZnO NPs, a slight broadening of this band is evident. However, this broadening is not observed for ZnO QDs. This suggests that the vibrational responses of the QDs are likely suppressed by the surrounding polymeric matrix. These findings indicate that the QDs are more uniformly dispersed or are more deeply embedded within the bulk of the PHB matrix, resulting in diminished spectral contributions.

**Figure 5:**
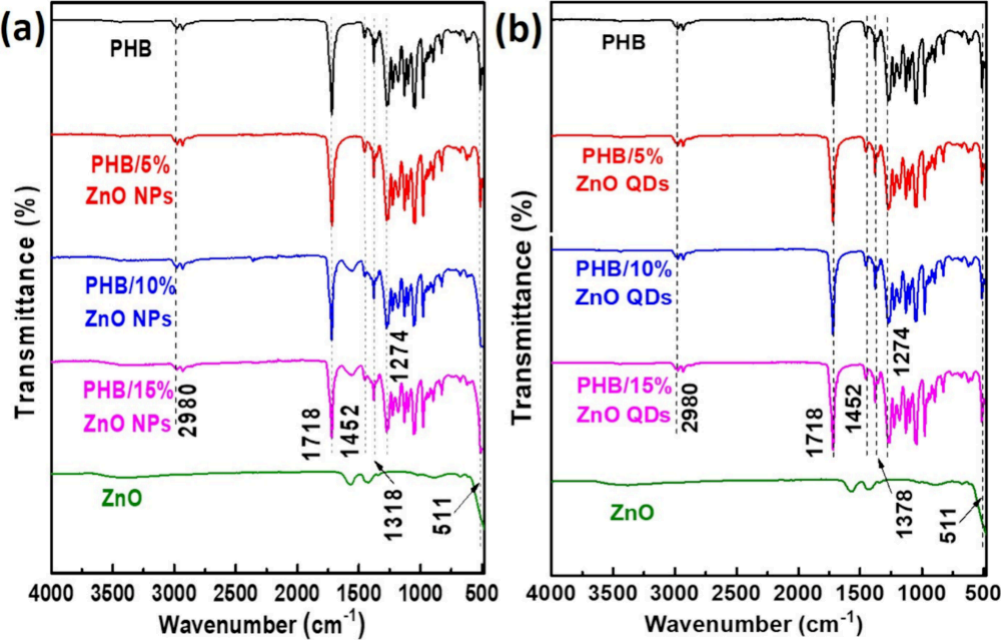
FTIR spectra of PHB membranes incorporated with different amounts of ZnO NPs (a) and QDs (b).

The UV-blocking capabilities of PHB/ZnO NPs and PHB/ZnO QDs membranes were assessed by exposing them to UV light for 45 days in a box. FTIR spectroscopy was used to analyze the samples, and spectral changes were evaluated using the intensity ratio method, which compares the ratio between specific pairs of characteristic bands (band 1:1719 cm^–1^ and band 2:1453 cm^–1^) before and after exposure to UV light. Figure 6 shows the FTIR spectra of the neat PHB membrane and membranes containing 15% ZnO NPs or QDs before and after UV exposure. Spectra for other ZnO loadings are provided in Figure S1. Table 1 presents the intensity ratio values of two characteristic bands for all NPs and QDs loadings analyzed, as well as for the membrane composed entirely of PHB.

**Figure 6:**
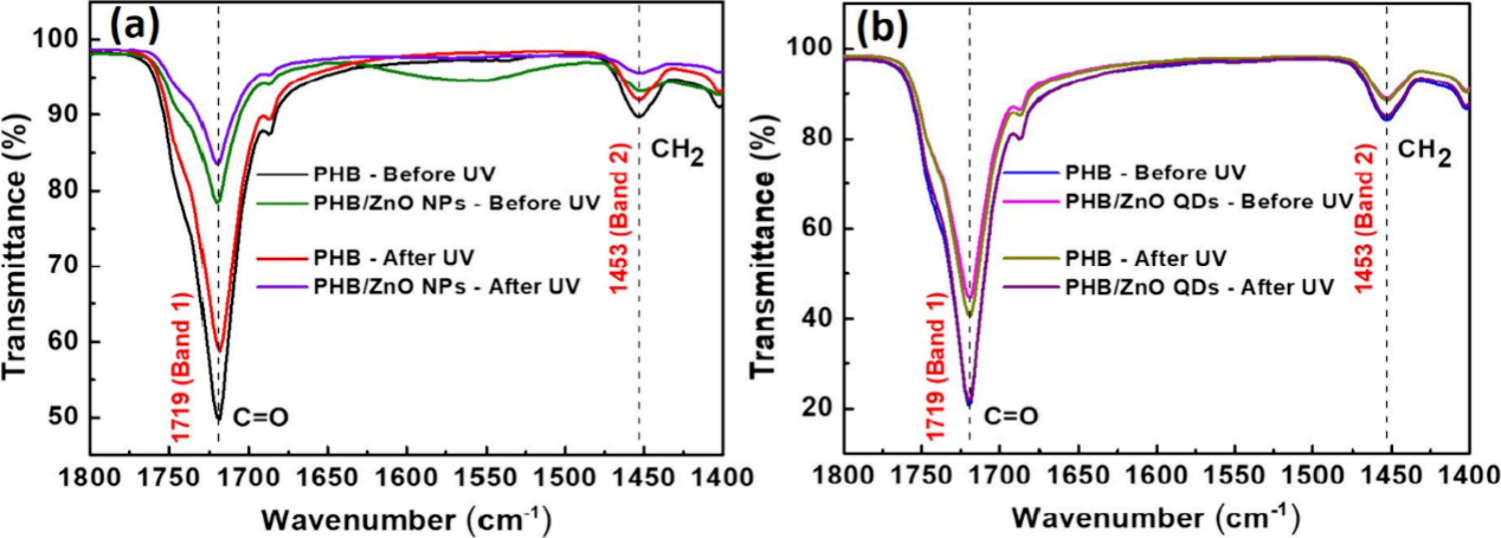
FTIR spectra of membranes containing 15% ZnO (NPs/QDs) before and after UV light exposure: PHB/ZnO NPs (a) and PHB/ZnO QDs (b).

**Table 1:** Ratio of Band Intensities (1 and 2) in Infrared (FTIR) Spectra of Neat PHB, PHB with ZnO NPs, and PHB with QDs Membranes before and after UV Exposure

membranes	charge (%)	before Band 1	before Band 2	ratio $\frac{1}{2}$	after Band 1	after Band 2	ratio $\frac{1}{2}$
PHB	**0**	49.30	9.640	5.114	40.58	7.374	5.503
PHB/ZnO NPs	**5**	23.23	4.846	**4.793**	80.04	15.54	**5.151**
	**10**	15.88	4.161	**3.816**	14.40	2.924	**4.925**
	**15**	21.20	6.558	**3.233**	15.77	4.293	**3.673**
PHB	**0**	78.68	10.53	**7.472**	76.33	10.13	**7.535**
PHB/ZnO QDs	**5**	74.11	14.30	**5.183**	78.82	15.17	**5.196**
	**10**	23.57	4.733	**4.979**	74.85	14.63	**5.116**
	**15**	54.97	15.67	**3.508**	57.85	13.60	**4.254**

Overall, the results indicate that increasing the loading of ZnO NPs and QDs led to a reduction in the intensity ratio between characteristic bands before UV exposure, a trend that remained consistent after exposure. For instance, the intensity ratios (before exposure) between bands 1 and 2 for neat PHB and PHB with 15% ZnO NPs were 5.114 and 3.233, respectively. Otherwise, after exposure, the intensity ratios were 5.503 and 3.673, respectively. These findings suggest that ZnO nanostructures interact with the carbonyl groups of the PHB matrix, enhancing the polymer’s resistance to photodegradation. This interaction improves the material’s durability and extends its potential applications. The UV-blocking effect arises from the ability of ZnO NPs and QDs to absorb ultraviolet radiation and convert it into harmless infrared energy, which is dissipated as heat,^44^ effectively forming a protective barrier against solar radiation.

#### 3.3.3 Remission Spectrophotometric

The UV radiation-blocking capacity of the composite membranes was confirmed through remission spectroscopy, which detects color changes in the material. To assess this property, the membranes were exposed to UV light in a box for 45 days. The results of this analysis are presented in Figure 7.

**Figure 7:**
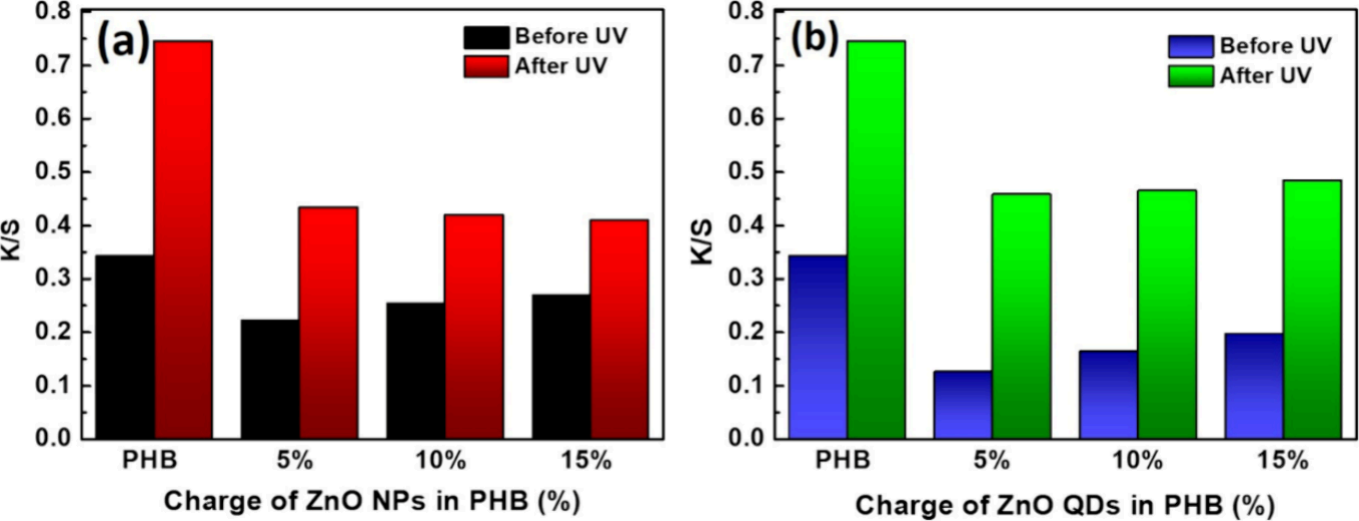
Remission spectrophotometric analysis of PHB membranes before and after 45 days of exposure to UV light (26 W): (a) PHB/ZnO NPs and (b) PHB/ZnO QDs.

The responses obtained for both membranes (Figure 7a,b) indicate that the neat PHB membrane presented a high degree of yellowing. This correlation is expected since K/S represents the coloristic intensity. Furthermore, a significant color difference of Δ*E* = 0.402 indicates a substantial change in the color intensity. This behavior suggests that the PHB membrane underwent oxidation or degradation under exposure to UV light. In addition, it was found that the membrane containing ZnO NPs presented a UV-blocking capacity better than that of the membrane containing QDs. It can also be observed that the higher the ZnO NPs charge, the higher the blocking efficiency. The property of blocking UV light enhances the application of PHB with ZnO NPs and QDs in products that shield UV light, making them more durable and resistant.^45^ The observed spectroscopic shifts correlate with the improved UV-blocking performance and structural stability of the PHB/ZnO membranes. UV–vis and fluorescence data confirm enhanced light absorption and emission, which is relevant for photoprotection and biomedical applications. FTIR results indicated interactions between ZnO and PHB carbonyl groups, reducing photodegradation and increasing durability, key advantages for packaging and biomedical fields.^45^

Numerous studies have demonstrated that ZnO NPs exhibit UV shielding through a combination of absorption and scattering mechanisms, which are intrinsically linked to their nanoscale optical properties and ability to prevent UV penetration.^46,47^ Beyond passive protection, ZnO NPs actively absorb UV photons, promoting electrons from the valence to the conduction band and generating electron–hole pairs that initiate photocatalytic reactions, particularly via the formation of reactive oxygen species (ROS) in the presence of water or dissolved oxygen.^48^ Importantly, this photocatalytic behavior can persist even when ZnO is immobilized within polymeric matrices,^49^ suggesting that ROS generation could potentially interact with the surrounding polymer. This interaction may lead to oxidative modifications of the polymer chains, potentially altering surface properties such as hydrophilicity.^47^

### 3.4 Analysis of the Physicochemical Properties of Membranes

Fluorescence stability tests were performed in a closed system under a humidity-saturated environment. Stability was evaluated qualitatively for 180 days by the incidence of UV light with an excitation at 365 nm. As shown in Figure S2, water vapor did not influence the visual fluorescence emission of the solid PHB film containing 15% ZnO NPs. Furthermore, it was verified that there was no variation in the mass of the material over this time interval.

Table 2 presents the density assessment of PHB membranes compared to PHB membranes incorporating increasing contents of NPs and ZnO QDs, along with the statistical analysis of the test data. It can be seen that the average value obtained for the density of neat PHB is 1.370 ± 0.010 g cm^–3^, a value close to that already mentioned in the literature.^50^ The statistical evaluation verified that there was no statistically significant difference between PHB + 5% ZnO QDs in the density values (same letter (A) in the table) with the PHB sample. However, when the analysis is performed between the groups of all samples, PHB/10% ZnO NPs do not present a statistically significant difference in density compared to 5% and 15% ZnO NPs (same letters in table (B,C)). The other samples showed a statistically significant difference between them, with a value of *p* ≤ 0.05. The results showed that adding NPs changes the membrane density for all ZnO loadings compared with PHB. The difference between neat PHB and the ZnO-loaded membranes is less pronounced for incorporating QDs, with no statistically significant difference observed for PHB/5% ZnO QDs.

**Table 2:** Density of Composite Membranes with Different Amounts of ZnO QDs and NPs and Neat PHB^a^

membranes	charge (%)	density (g cm^–3^)
PHB		1.370 ± 0.090^(A)^
	5	1.404 ± 0.162^(B)^
PHB/ZnO NPs	10	1.413 ± 0.168^(B,^ ^C)^
	15	1.420 ± 0.174^(C)^
	5	1.378 ± 0.082^(A)^
PHB/ZnO QDs	10	1.387 ± 0.084^(D)^
	15	1.395 ± 0.087^(E)^

a Equal letters indicate no statistically significant difference (*t-*test with 95% confidence level). Uppercase and lowercase letters correspond to the interpretation of the Student’s *t-*test and the associated *p*-value.

Furthermore, it was also found that the different ZnO nanostructures (QDs and NPs) accommodated themselves differently in the matrix, establishing an interaction between the polymer and ZnO, reducing vacancies, and strengthening the polymer structure. In a study with PHB composite, it was observed that with the addition of rice husk ash (RHA) loads, the PHB/RHA composites underwent a modest and linear increase in density.^51^

Table 3 shows the contact angle measurements (average values) for PHB membranes and those containing different amounts of ZnO NPs and QDs after 1 and 8 min of interaction with a water droplet. These measurements are essential to obtain information about the membrane surface, such as wettability. As can be seen, all membranes showed hydrophilic behavior (contact angle < 90°), which shows that the water droplet spreads after adhering to the membrane surface.^52^ It can also be observed that there was no significant difference in contact angle between the neat PHB membrane (61.10° ± 1.90°) and the membrane with 5% NPs (60.56° ± 0.49°) after 1 min of interaction. Although the angle decreased after 8 min, the behavior between them remained the same: when the percentage of NPs increased to 10% and 15%, the angle value decreased to 55.53° ± 0.53° and 53.49° ± 0.55°, respectively, making the membranes more hydrophilic. Figure S3 presents images of the water droplet formed on the surface of the membranes after 1 min.

**Table 3:** Results of Contact Angles Determined after 1 and 8 Min of Interaction of the Water Droplet with the PHB Membranes and Those Modified with Different Contents of QDs and ZnO NPs

membranes	charges (%)	contact angle (°) 1 min	contact angle (°) 8 min
PHB	0	61.10 ± 1.90^(A,^ ^a)^ [Table-fn t3fn1]	58.18 ± 2.53^(A,^ ^a)^
	5	60.56 ± 0.49^(A,^ ^a)^	57.68 ± 0.52^(A,^ ^b)^
PHB/ZnO NPs	10	55.53 ± 0.53^(B,^ ^a)^	51.84 ± 0.57^(B,^ ^b)^
	15	53.49 ± 0.55^(C,^ ^a)^	50.72 ± 0.59^(B,^ ^b)^
	5	52.35 ± 0.57^(C,^ ^a)^	48.24 ± 0.60^(C,^ ^b)^
PHB/ZnO QDs	10	42.70 ± 0.59^(D,^ ^a)^	40.88 ± 0.62^(D,^ ^b)^
	15	40.42 ± 0.61^(E,^ ^a)^	36.07 ± 0.64^(E,^ ^b)^

* Capital letters: These are different samples, but at the same time, lowercase letters are the same sample at other times. The same letters mean no statistically significant difference (*t*-test with 95% confidence level). Uppercase and lowercase letters correspond to the interpretation of the Student’s *t*-test and the associated *p*-value.

The water droplet spreads more pronouncedly on the surface of the PHB membrane with ZnO QDs. This statement can be further verified in Table 3, where the contact angle values calculated for this membrane were lower than those obtained for the membranes with the exact amounts of ZnO NPs. This result can be explained by the difference in particle size, with ZnO QDs being in the range ∼ 4 nm, while NPs have an average size of 10 nm. Thus, the QDs present a larger specific surface area and can interact better with the polymer matrix and the water droplet, making the material more hydrophilic compared with the equivalent charge of ZnO NPs.

The membrane performance was also evaluated by hardness (Shore A), swelling, and permeability tests; the results of these analyses are shown in Figure 8. Figure 8a reveals a significant increase in membrane hardness after incorporation of ZnO NPs and QDs. It is also observed that there is a slight increase in hardness with the increase in the content of NPs and QDs, showing an improvement in the resistance of the membranes, even with a low percentage of loading. This property, presented by the composite membranes, can increase their degree of future applicability. These results align with previous studies, which demonstrate an increase in material hardness following modification. For instance, Barletta and collaborators^53^ presented a compression-molded PHB film whose hardness was increased after the addition of graphene nanoplatelets (GNPs) and amino-functionalized nanosilica (A-fnSiO_2_) to the polymer. Also, Fernandes et al.^54^ showed that the increase in hardness of the PHB/quasi-crystals (poly­(hydroxybutyrate/Al_62_._0_Cu_25.5_Fe_12.5_) is proportional to the increase in inorganic charge.

**Figure 8:**
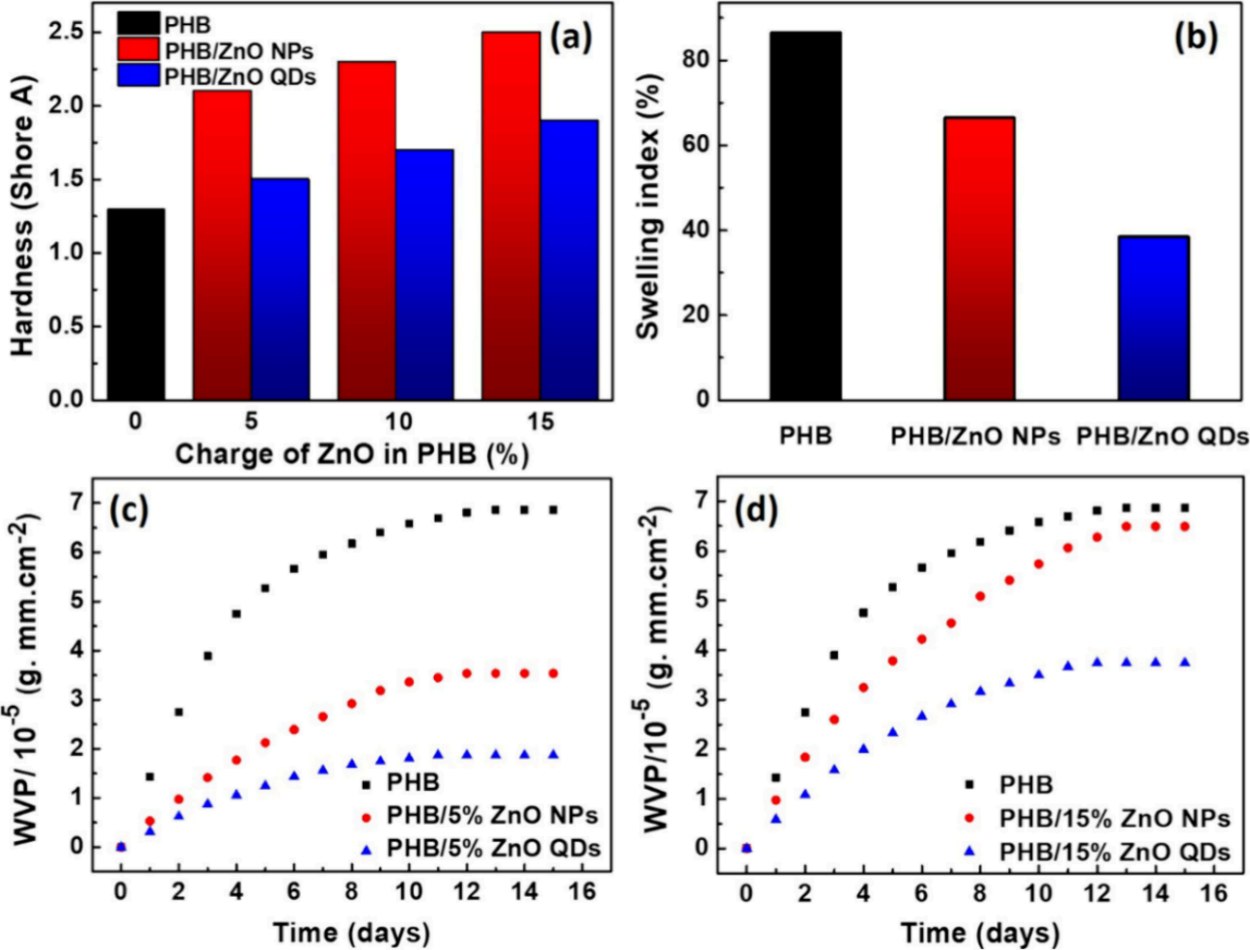
Results of the hardness test (shore A) of the PHB membrane and composite membranes (a), of the swelling index (sample with 15% loading of ZnO NPs and QDs) (b), and the water vapor permeability (WVP) test (membranes with 5% (c) and 15% (d) of ZnO NPs and QDs).

The swelling properties of the PHB membranes and those with 15% NPs and QDs loading were analyzed to verify changes in water absorption upon the incorporation of nanostructures. The results of this analysis are shown in Figure 8b. It can be observed that there was a mass gain for the neat PHB membrane of up to 86.6% after 12 h of contact with water, a value that remained constant after 24 h. The reduction in the capacity of the membrane to retain water was noted after the addition of ZnO NPs and QDs to the polymer matrix. Membranes with 15% NPs and QDs for the same period (12 h) had a mass gain of 66.5% and 38.4%, respectively. In earlier observations, the incorporation of RHA into the PHB matrix was found to significantly enhance the swelling index. Specifically, when RHA was added as an inorganic filler at concentrations of 10% and 15% by weight, the swelling percentage increased from 55% for neat PHB to 72% and 77%, respectively. This indicates a substantial impact of RHA on the material’s swelling behavior, different from the present study.^9^

The results suggest a hydrogen bonding interaction between the PHB molecule and water, facilitating mass gain. A strictly bonded network is formed after incorporating ZnO NPs and QDs, reducing the intermolecular space between the chains and making membrane interaction and water penetration difficult. This behavior was also observed in a previous study by Schmitz and his collaborators.^38^ The authors showed that after adding ZnO NPs and QDs to the zein biopolymer matrix, there was a reduction of up to three times in water absorption compared to zein. Thus, incorporating QDs and ZnO NPs allows new arrangements between the nanostructures and the polymer.

Figure 8 also shows the results of the WVP test after 15 days of monitoring. This test is essential to evaluate the moisture barrier capacity of materials, mainly those used in the food industry, such as food packaging.^55,56^

The PHB and PHB membranes containing 5% (Figure 8c) and 15% (Figure 8d) ZnO NPs and QDs were evaluated. As can be seen, as the days go by, there is a progressive increase in the amount of water vapor absorbed by the membranes. From the fifth day onward, this amount tends to equilibrium, when water vapor will no longer be absorbed, except for the membrane with 15% of ZnO NPs, in which the tendency toward saturation begins on the ninth day. It can also be noted that the neat PHB sample showed a more significant increase compared to the modified membranes. The maximum WVP values for PHB and PHB/15% ZnO NPs were 6.86 × 10^–5^ and 6.48 × 10^–5^ g mm cm^–2^, respectively. The minimum WVP values were 3.56 × 10^–5^ (PHB/5% ZnO NPs) and 1.87 × 10^–5^ g mm cm^–2^ (PHB/5% ZnO QDs), presenting a reduction of 48.10% and 72.74%, respectively. Therefore, although the PHB membrane is superficially more hydrophilic after the insertion of NPs and QDs, the results in the WVP reduction showed that these nanostructures can form barriers to water penetration, not favoring the diffusion of water molecules. In this sense, optimizing membrane properties to achieve lower vapor permeability may lead to tailored packaging solutions for specific food types, enhancing their protective qualities and potentially expanding their applications in sectors such as pharmaceuticals and cosmetics, where moisture control is critical for reducing food waste and improving sustainability in the production supply chain. The apparent paradox between increased surface hydrophilicity and reduced WVP can be explained by molecular-level interactions. ZnO NPs and QDs enhance surface wettability due to their high surface area and polar nature, with the effect being more pronounced for QDs. Density measurements suggest that incorporation into the PHB matrix promotes a denser polymer network through specific intermolecular/particle bonding and physical cross-linking. This tighter structure reduces the free volume and limits pathways for water vapor diffusion, effectively enhancing the barrier properties. A schematic illustrating these interactions to clarify this mechanism is shown in Figure 9.

**Figure 9:**
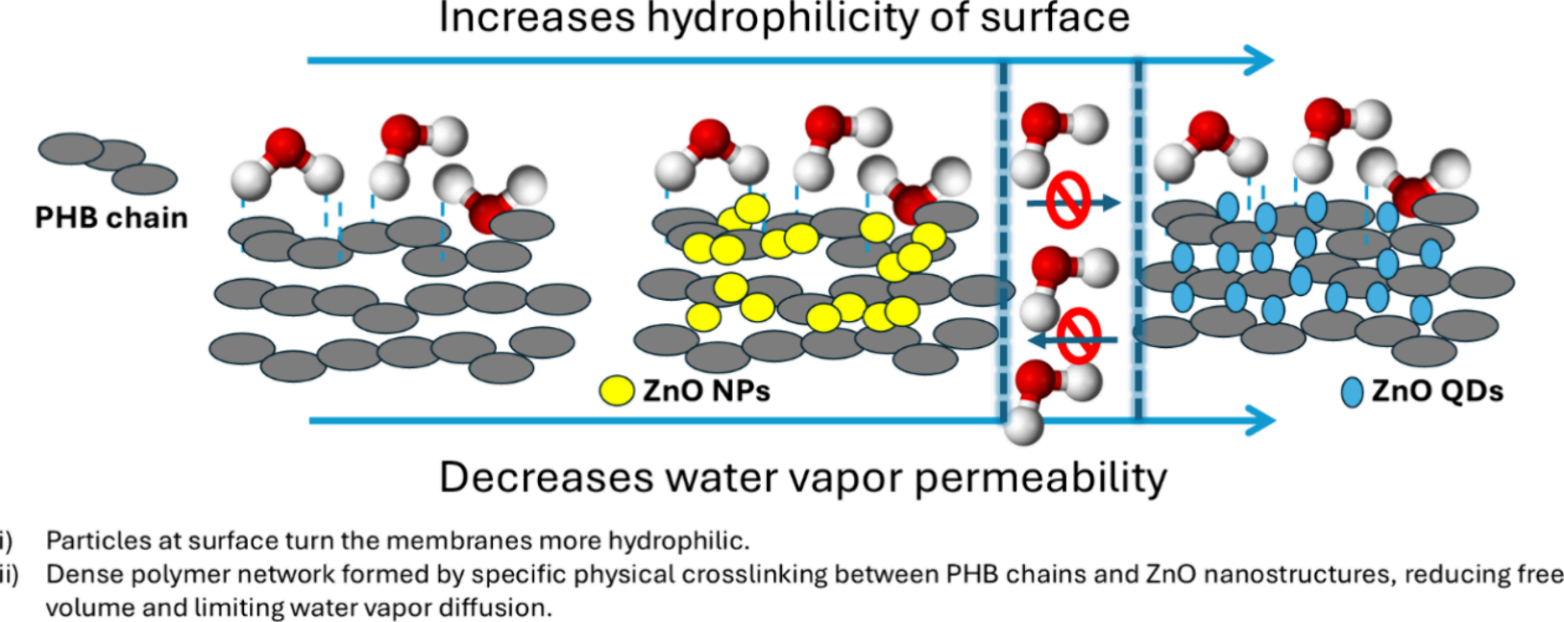
Schematic representation of PHB and PHB composites containing ZnO QDs and NPs, highlighting their influence on the hydrophilicity and water vapor permeability.

Through TGA, it was not possible to infer the same nominal mass contents in the residual portion following the thermal decomposition of PHB. Although the overall degradation profiles of the PHB-based samples remained largely consistent with those of neat PHB, the presence of ZnO nanostructures introduced subtle changes in thermal behavior. The similarity in the thermogram shapes suggests that the primary decomposition mechanism of PHB was not significantly altered by the incorporation of ZnO. However, the onset of thermal degradation occurred at a slightly lower temperature in the ZnO-containing samples, which implies that ZnO may facilitate earlier chain scission or promote thermal instability at lower temperatures, possibly due to interactions at the polymer–NP interface. Specifically, the sample with 10% ZnO NPs exhibited an onset degradation temperature of 259 °C, compared to 272 °C for neat PHB. Despite this earlier onset, the single-step degradation pattern and the position of the inflection point (observed through the first derivative analysis—not shown) remained relatively unchanged, supporting the idea that the fundamental thermal decomposition pathway of PHB is preserved (see Figure S4).

### 3.5 Applications of Membranes

#### 3.5.1 Antimicrobial Activity

The composite membranes (with 5% and 15% charge) were evaluated for inhibition of the growth of *S. aureus* (Gram-positive) bacteria; the results are shown in Table 4. The literature reports that PHB has no antimicrobial activity.^57,58^ On the other hand, ZnO NPs and QDs are known to have excellent results in this application.^59^ The mechanism of antibacterial activity of ZnO NPs/QDs is based on structural changes in the microbial cell membrane and cytoplasmic leakage or the depletion of cellular constituents that can lead to the death of bacterial cells.^60,61^ Production of ROS was investigated as the main mechanism of ZnO nanostructures (NPs, nanorods, and hierarchical flower-like) toward Gram-positive and Gram-negative antibacterial activity. *S. aureus* was more sensitive to the structure's effect, and the number of bacteria decreased in a concentration-dependent manner.^62^ Also, the antibacterial activity of ZnO can be related to ZnO NPs internalization and Zn^2+^ release.^63^ Table 4 shows that membranes with ZnO NPs increased the bacterial inhibitory effect (15% ZnO NPs: 30.08 ± 3.07) compared to PHB. Furthermore, membranes containing ZnO QDs showed the opposite behavior of the NPs, with increasing charge, and there was a decrease in the inhibition of the growth of the bacterial *S. aureus*. The smaller size of the QDs, and consequently their larger surface area, should have resulted in a higher percentage of bacterial inhibition with increasing concentration;^62^ however, this was not observed. It is therefore suggested that a majority of QDs are located within the inner regions of the PHB matrix or embedded in the bulk of the membrane, which may hinder their interaction with bacteria. Supporting this interpretation, FTIR analysis for PHB membranes did not reveal the Zn–O vibration band for QDs, unlike the signal observed for ZnO NPs. An additional inference is that the incorporation of QDs had a more pronounced impact on surface wettability compared to NPs, as evidenced by a greater reduction in contact angle (Table 3). This suggests that QDs significantly increase the surface energy. According to literature,^64^ higher surface energy tends to enhance bacterial adhesion, thereby promoting biofilm formation, which would justify the smaller effect for 15% QDs compared to the same content of ZnO NPs.

**Table 4:** Antimicrobial Activity against *S. aureus* of Neat PHB and PHB/ZnO NPs and QDs Membranes

membranes	charge (%)	% inhibition
		*S. aureus*
PHB		(2.98 ± 1.02)^A^ [Table-fn t4fn1]
PHB/ZnO NPs	5	(20.97 ± 3.79)^B^
	15	(30.08 ± 3.07)^C^
PHB/ZnO QDs	5	(21.83 ± 4.95)^B^
	15	(2.51 ± 0.34)^A^

* The same letters mean no statistically significant difference (*t*-test with 95% confidence level). Uppercase and lowercase letters correspond to the interpretation of the Student’s *t*-test and the associated *P*-value.

Primary antimicrobial tests against *Escherichia coli* (Gram-negative) were conducted by our research group,^38^ using ZnO NPs and QDs embedded in zein. Both nanostructured systems exhibited a stronger inhibitory effect against *Staphylococcus aureus*, while no additional inhibitory activity beyond that of the zein matrix was observed against *E. coli* when tested with ZnO NPs or ZnO QDs.

#### 3.5.2 Photocatalytic Activity

The degradation of the Astrazone Blue (AB) dye was investigated under a sunlight lamp (300 W) using a PHB membrane incorporated with 15% ZnO NPs. Before that, the effect of light on the degradation of the dye without the presence of a membrane was evaluated. The results of this analysis can be seen through the spectra of the dye presented in Figure 10. It is observed that for both concentrations (7.5 × 10^–6^ mol L^–1^ (Figure 10a) and 1.5 × 10^–5^ mol L^–1^ (Figure 10b)) evaluated, there was no significant decay of the maximum absorbance band (609 nm), even after 4 h of monitoring, showing that light alone does not degrade the dye. The same procedure was used for a neat PHB membrane, and the result showed that there was no significant decay of the maximum absorbance band of the dye.

**Figure 10:**
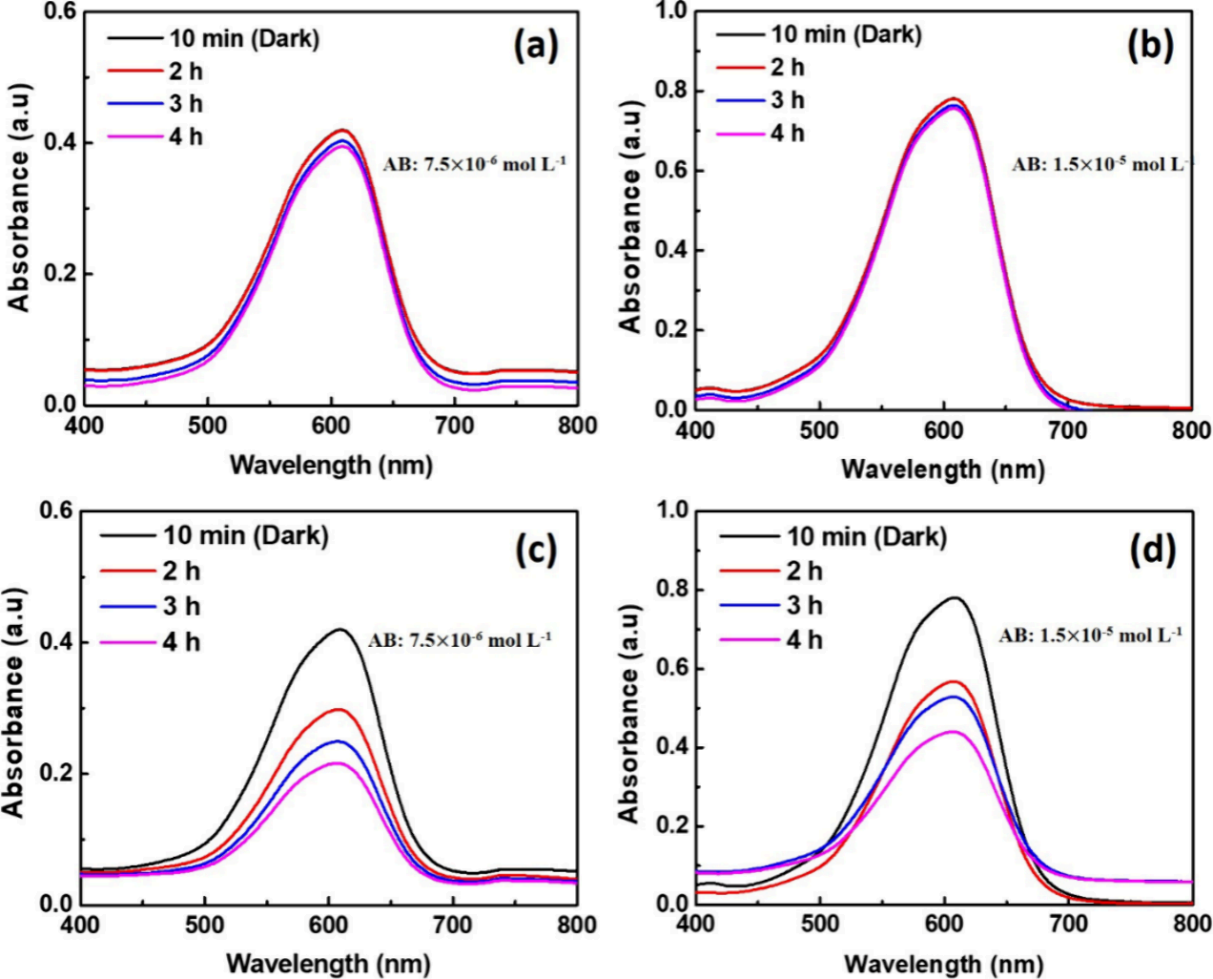
Absorbance spectra after the degradation test of the Astrazon Blue (AB) dye at different concentrations only under the incidence of light (a) 7.5 × 10^–6^ mol L^–1^ and (b) 1.5 × 10^–5^ mol L^–1^ and after interaction with the PHB/15% ZnO NPs membrane (c) 7.5 × 10^–6^ mol L^–1^ and (d) 1.5 × 10^–5^ mol L^–1^.

On the other hand, in the dye discoloration tests in the presence of the membrane (PHB/15% ZnO NPs), there was a decay of absorbance with time for both concentrations, as can be observed in Figure 10c (7.5 × 10^–6^ mol L^–1^) and d (1.5 × 10^–5^ mol L^–1^). After 4 h, the degradation percentage was 48.35% and 48.78% for the concentration of 7.5 × 10^–6^ and 1.5 × 10^–5^ mol L^–1^, respectively (correcting the baseline effect). The results suggest that the photocatalytic performance was sustained over the same time scale for both tested concentrations. This indicates that the active photocatalyst supported on the membrane effectively degraded organic compounds dispersed in the liquid medium and that doubling the pollutant concentration did not lead to saturation of the photocatalytic sites.

Although the tests were designed as a preliminary evaluation, the findings indicate that incorporating photocatalytic agents into the membrane shows promise for small-scale interfacial reactions, as elsewhere reported about the uses and photocatalytic performance of ZnO composites.^65^ A key advantage of this approach is the immobilization of NPs, which are typically difficult to separate when in colloidal form, as is the case with ZnO QDs, to degrade AB in an ethanolic medium.^21^ The membrane system presented here offers a practical solution by embedding the photocatalytic activity within a solid matrix, thereby facilitating its separation from the reaction medium. This configuration could enable the development of self-cleaning surfaces capable of degrading organic molecules that accumulate directly on the membrane.

While recyclability and reusability were not assessed in the present study, we recognize their importance for practical applications. Future investigations will aim to evaluate the stability and retention of photocatalytic activity over multiple cycles, in order to determine the membrane’s long-term performance and feasibility for repeated use.

## 4 Conclusions

This study describes the preparation of a biodegradable polymer matrix of PHB incorporated with ZnO NPs and QDs. After 2 h of synthesis, UV–vis absorption spectra indicated the successful formation of NPs and QDs. Microscopic measurements revealed particle sizes of 6.3 nm for NPs and 4.3 nm for QDs (based on previous work). Stable and homogeneous ZnO NPs and QDs composite membranes were prepared with UV–vis and fluorescence spectrophotometry, confirming their presence. The results also showed increases in absorption and fluorescence intensity as the loading of nanostructures in the polymer matrix increased. FTIR spectra revealed characteristic bands of the polymer, and spectral changes were observed with the presence of NPs and QDs in the membrane. Colorimetric analysis of the membranes under UV light exposure confirmed that neat PHB exhibited a more pronounced yellowish hue than those with ZnO NPs and QDs during the 45-day evaluation period. The composite membranes also demonstrated a higher density, wettability, and hardness than the PHB membrane. Additionally, swelling analysis combined with WVP testing showed that adding nanostructures reduced water absorption compared with the unmodified biopolymer, with QDs having a more pronounced effect. These findings highlight a nuanced relationship between the surface hydrophilicity and WVP in PHB membranes modified with NPs and QDs. While the surface hydrophilicity increases, the reduction in WVP suggests that these nanostructures effectively hinder moisture diffusion by forming internal barriers. This paradox underscores the importance of not relying solely on surface properties in the assessment of functional performance. Strategic optimization of these nanocomposite membranes paves the way for designing advanced packaging materials that are suited for specific moisture-sensitive applications. Beyond food preservation, this approach shows promise in the pharmaceutical and cosmetic sectors, where moisture control is crucial for product stability, shelf life, and sustainability, ultimately aiding in reducing waste and enhancing supply chain efficiency.

Inhibitory activity tests against the growth of *S. aureus* were performed using composite membranes. The results showed that the materials are promising for this application, especially the membrane containing the ZnO NPs, where an increase in the NPs loading led to a significant increase in the inhibitory percentage. It was also possible to evaluate the photocatalytic action of PHB with 15% ZnO NPs. The membrane proved efficient for the photodegradation of the dye Astrazon Blue from the solid/liquid interface with a value of 48.35 and 48.78% for dye concentrations of 7.5 × 10^–6^ and 1.5 × 10^–5^ mol L^–1^, respectively.

The results described here demonstrate that the presence of ZnO NPs and QDs in different mass ratios in the PHB polymer matrix is promising for the design of new materials with improved properties. For example, food packaging provides better storage conditions, prevents the proliferation of fungi and bacteria, and protects household items against UV radiation. While the current study focuses on the UV shielding and structural integrity of the composite, further investigation is warranted to elucidate the long-term effects of UV exposure on both the ZnO NPs and the polymeric matrix. Such studies could provide deeper insights into the stability, performance, and potential degradation pathways of the composite materials under prolonged UV irradiation.

## Supplementary Material



## Notes

Equations for bandgap energy and membrane volume calculations; table of hydrodynamic size of QDs dispersions over 21-day storage (DLS); complete FTIR spectra of membranes before and after UV exposure; TGA analysis; photographs of fluorescence emission of PHB/ZnO NP membranes; photographs of water contact angle for PHB and PHB/ZnO membranes after 1 min interaction (PDF)
