# Supplementary material for: Tunable Nanoeffect of ZnO on the Properties of Poly(hydroxybutyrate) Membranes
Source: ACS Omega. 2025 Dec 3;10(49):60659–72. doi: 10.1021/acsomega.5c08548 (PMC12713433; doi:10.1021/acsomega.5c08548)
Supplement: Supplementary file 1 [file ao5c08548_si_001.pdf]

Supporting Information

Tunable Nanoeffect of ZnO on the Properties of Poly(hydroxybutyrate) Membranes

Tainan Miguel,[a] Winnie Queiroz Brandão, [a] Martinho Rau,[a], Michele Debiasi Alberton,[b] Ivonete O. Barcellos, [a] Lizandra Maria Zimmermann[a*]

[a] Department of Chemistry, Regional University of Blumenau - FURB, 89030-903 Blumenau, SC, Brazil

[b] Department of Pharmaceutical Sciences, Regional University of Blumenau - FURB, 89030-903 Blumenau, SC, Brazil

* Corresponding author

Tel: +55 047 3321 0276

E-mail: lmz@furb.br

1. *Characterization of NPs and QDs dispersions*

The absorption band in the UV-Vis region allows one to visualize the quantum confinement of the semiconductor particles and, thus, determine the size of the QDs through Eq. S1 [1, 2],

$$d = 2 \times \frac{-0.3049 + \sqrt{-26.23012 + \frac{10240.72}{\lambda_{Bg}}}}{-6.3829 + \frac{2483.2}{\lambda_{Bg}}} \qquad \text{Eq. S1}$$

where λ_{Bg} is the wavelength of the forbidden band, obtained from the beginning of the absorbance vs wavelength (nm) curve, the constants are ZnO characteristics.

The band gap energy was calculated using the Planck-Einstein relation (Eq. S2), where E_g represents the band gap energy (eV), *c* is the speed of light in vacuum and *h* is Planck's constant.

$$E_g = \frac{ch}{\lambda_{Bg}} \qquad \text{Eq. S2}$$

Stability of QDs dispersion over time was investigated by DLS, as shown in Table S1.

Table S1. Hydrodynamic diameter for ZnO QDs colloidal dispersions 2×10^{-3} mol L^{-1} over 21 days

**Time**	**Z- Average (d.nm)**
After synthesis	4.3
1 day	12.5
7 days	14.6
21 days	15.7

2. *Physicochemical Analysis*

2.1. *Density determination*

The density of the pure and modified PHB membranes was determined by their mass and volume ratio. The membrane mass was obtained, followed by measuring the dimensions with a digital micrometer and a Mitutoyo caliper. Using Eq. S3, the volume of the membranes was calculated.

$$V = \pi \, x \, r^2 x \, h \qquad \text{Eq. S3}$$

Where r (cm) is the membrane's radius, the diameter was first measured with a Mitutoyo caliper to determine this. Finally, the membrane's thickness h (cm) was measured with a digital micrometer.

3. *Fourier Transform Infrared Spectroscopy (FTIR)*

The pure and modified PHB membranes were left under ultraviolet light for 45 days in a dark chamber and then characterized by FTIR. The results are shown in Fig. S1.

Fig. S1. Infrared spectra before and after UV light exposure for neat PHB membrane and PHB incorporated with ZnO NPs (a and c) and neat PHB and PHB incorporated with ZnO QDs (b and d), respectively.

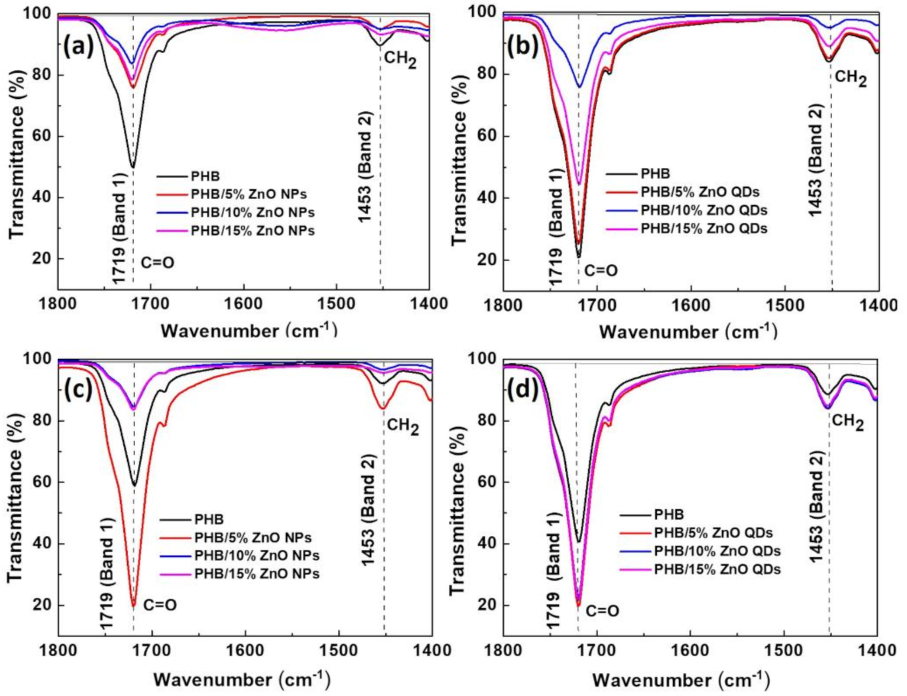

As shown in Fig. S2, water vapor did not influence the visual fluorescence emission of the solid PHB film containing 15% ZnO nanoparticles.

Fig. S2. Photographs of PHB membrane with 15% of ZnO NPs before exposure to humidity (a) and after 180 days (b), under a UV light lamp with excitation at 365 nm**.**

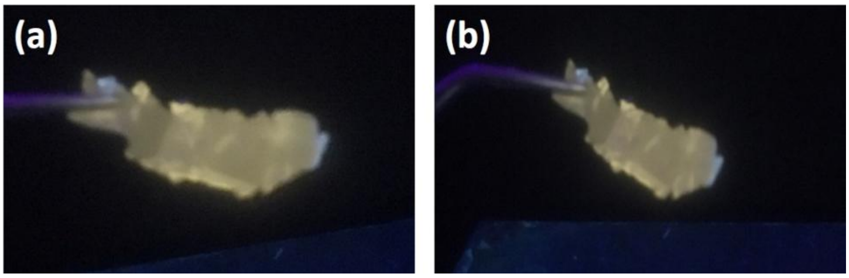

4. *Determination of contact angle*

The images of the water droplet on the surface of the pure PHB and PHB membranes with NPS and ZnO QDs were obtained by the USB Digital Microscope, with magnification up to 1000x. The ImageJ program was used to determine the value of the contact angle. The measurements were performed in triplicate, and the average was calculated. The results for 1 min of the droplet's interaction with the membranes are shown in Fig. S3.

Fig. S3: Images of the water droplet formed on the surface of the membranes and contact angle medium values calculated after 1 min of interaction. Set of images (I, II, III, IV): for neat PHB and ZnO NPs with increasing content. (a) for neat PHB and ZnO QDs with increasing content. (b)

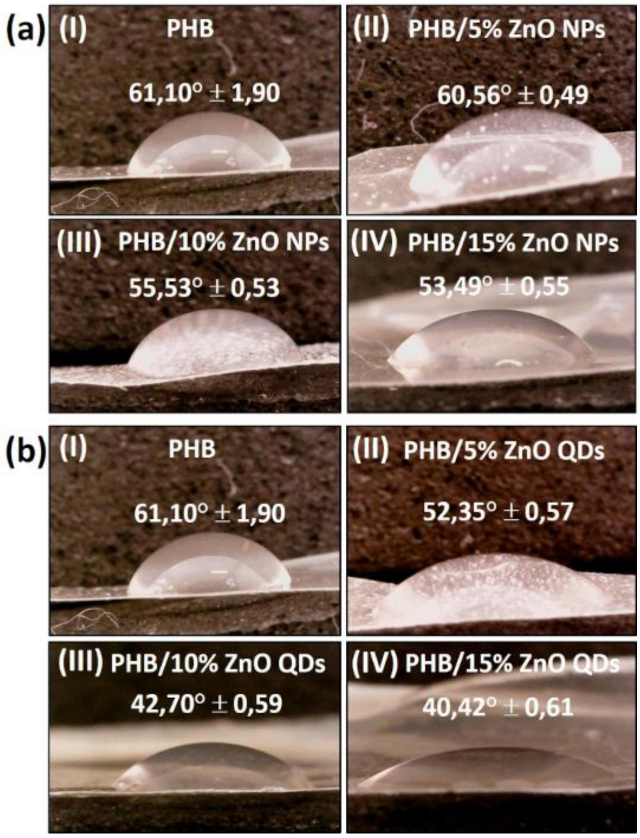

5. *Thermogravimetric Analysis*

Thermogravimetric analysis (TGA) measurements were carried out using a PerkinElmer 4000 instrument under a synthetic air atmosphere at a flow rate of 20 mL min^{-1}. The temperature was ramped from 30 to 800 °C at a heating rate of 10 °C min^{-1}. Fig. S4 presents selected sample curves, including an inset showing a magnified view of the specified temperature range.

Fig. S4: TGA analysis of four samples: pure PHB (———), PHB with 5% in weight ZnO QDs (———) and NPs (———), and with 10% ZnO NPs (———). Inset: Same samples with a new magnification scale.

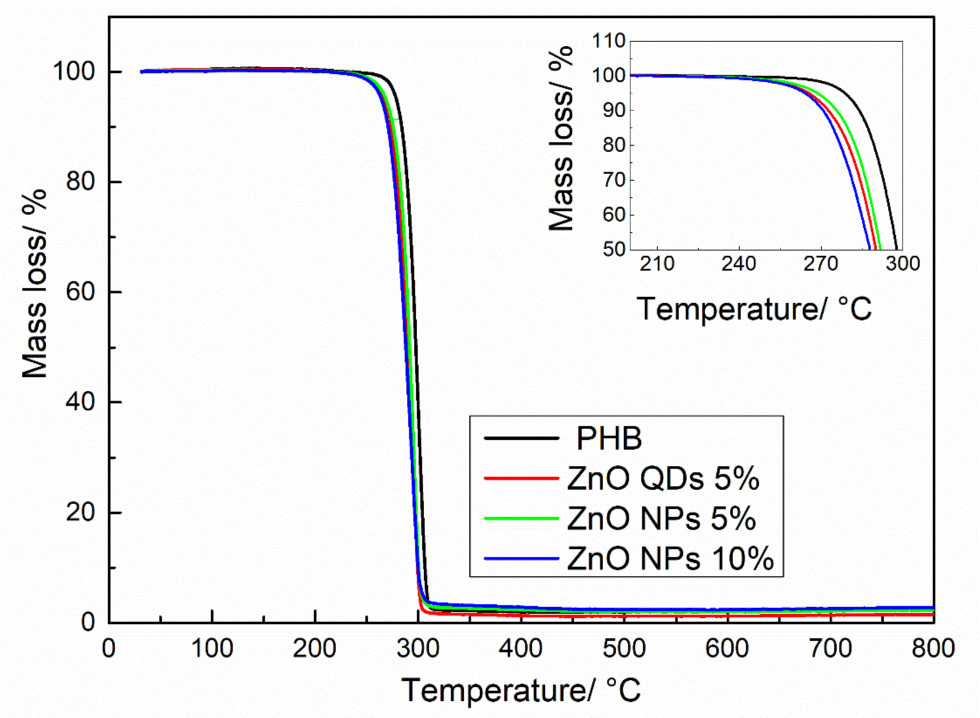

References

1. Brus, L.E., *Electron–electron and electron-hole interactions in small semiconductor crystallites: The size dependence of the lowest excited electronic state.* The Journal of chemical physics, 1984. **80**(9): p. 4403-4409.

2. Schmitz, F., et al., *Zein films with ZnO and ZnO: Mg quantum dots as functional nanofillers: New nanocomposites for food package with UV-blocker and antimicrobial properties.* Polymer Testing, 2020. **91**: p. 106709.
